# Multiple dietary supplements do not affect metabolic and cardiovascular health

**DOI:** 10.18632/aging.100597

**Published:** 2013-09-04

**Authors:** Andreea Soare, Edward P. Weiss, John O. Holloszy, Luigi Fontana

**Affiliations:** ^1^ Division of Geriatrics and Nutritional Sciences, Department of Medicine, Washington University School of Medicine, St. Louis, MO 63130, USA; ^2^ Department of Endocrinology and Diabetes, University Campus Bio-Medico, Rome, Italy; ^3^ Department of Nutrition and Dietetics, St. Louis University, St. Louis, MO 63130, USA; ^4^ Department of Medicine, Salerno University School of Medicine, Salerno, Italy; ^5^ CEINGE Biotecnologie Avanzate, Napoli, Italy

**Keywords:** supplements, endothelial function, arterial stiffness, inflammation, oxidative stress

## Abstract

Dietary supplements are widely used for health purposes. However, little is known about the metabolic and cardiovascular effects of combinations of popular over-the-counter supplements, each of which has been shown to have anti-oxidant, anti-inflammatory and pro-longevity properties in cell culture or animal studies. This study was a 6-month randomized, single-blind controlled trial, in which 56 non-obese (BMI 21.0-29.9 kg/m^2^) men and women, aged 38 to 55 yr, were assigned to a dietary supplement (SUP) group or control (CON) group, with a 6-month follow-up. The SUP group took 10 dietary supplements each day (100 mg of resveratrol, a complex of 800 mg each of green, black, and white tea extract, 250 mg of pomegranate extract, 650 mg of quercetin, 500 mg of acetyl-l-carnitine, 600 mg of lipoic acid, 900 mg of curcumin, 1 g of sesamin, 1.7 g of cinnamon bark extract, and 1.0 g fish oil). Both the SUP and CON groups took a daily multivitamin/mineral supplement. The main outcome measures were arterial stiffness, endothelial function, biomarkers of inflammation and oxidative stress, and cardiometabolic risk factors. Twenty-four weeks of daily supplementation with 10 dietary supplements did not affect arterial stiffness or endothelial function in nonobese individuals. These compounds also did not alter body fat measured by DEXA, blood pressure, plasma lipids, glucose, insulin, IGF-1, and markers of inflammation and oxidative stress. In summary, supplementation with a combination of popular dietary supplements has no cardiovascular or metabolic effects in non-obese relatively healthy individuals.

## INTRODUCTION

Non-vitamin, non-mineral dietary supplements are widely used for health purposes and sometimes as a substitute to a healthy diet or conventional medical treatments. Nearly 1 in 7 adults takes supplements regularly, and approximately 40% have taken one or more dietary supplements during their life [1]. However, despite the widespread and growing use of these over-the-counter products, insight into the potential beneficial or harmful biological effects of these compounds in humans is frequently lacking.

Some individuals, especially heavy supplement users, typically consume combinations of dietary supplements because they believe that multiple compounds can act through complimentary, additive or synergistic mechanisms to convey a greater biologic effect than can be achieved by any individual supplement [2]. The present study was a randomized clinical trial to evaluate the effectiveness of supplementation with a combination of some of the most self-prescribed dietary supplements (i.e. resveratrol, curcumin, green/black/white tea extract, quercetin, acetyl-l-carnitine, lipoic acid, pomegranate, cinnamon bark, sesamin, and fish oil), in lean and overweight middle-aged men and women eating a Western diet. It has been reported that these compounds exert powerful protective effects against inflammation, oxidative stress/free radical damage, insulin resistance, and protein glycation in cell culture and laboratory animal studies [3-24]. We evaluated the combined effects of these supplements on arterial stiffness, endothelial function, markers of inflammation, oxidative stress, glucose and lipid metabolism, and blood pressure.

## RESULTS

### Study Participants

**Figure 1:**
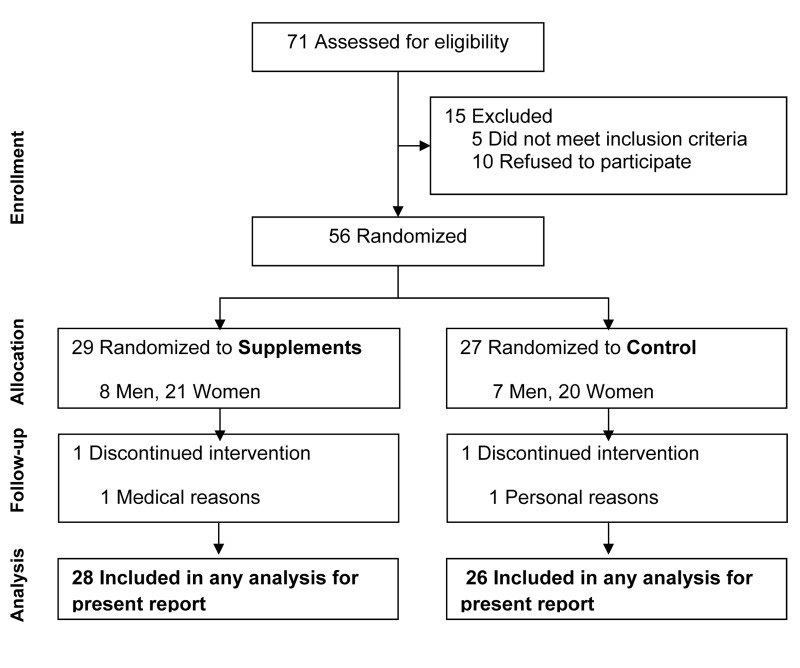
Consort diagram reflecting flow of study participants through the study

Screening, enrollment, and follow-up information is presented in Fig. 1. The study participants were generally healthy, as reflected by the following: BMI, 25.0±2.3 kg/m^2^; total cholesterol, 187±32 mg/dL; triglycerides, 79±53 mg/dL; fasting glucose, 82±9 mg/dL; and systolic and diastolic blood pressures of 109±12 and 68±8 mmHg, respectively. The proportions of men and women did not differ between groups (p=0.65). The average age of participants was slightly lower in the SUP group (means±SD: 44±6 vs. 47±5 yr, p=0.05). BMI did not differ (p=0.65) between the SUP (25.2±2.0 kg/m^2^) and control (24.9±2.5 kg/m^2^) groups.

Based on monthly pill compliance queries, 96% of the participants in the SUP group took all of the prescribed doses of the supplements. All participants in both groups complied with the vitamin/mineral supplementation regimen.

There were no differences between groups in PWV, AI, blood pressure, and endothelium-dependent and endothelium-independent brachial artery vasodilation (Table 1). No changes in body weight or % total body fat occurred in either study group (Table 2). Plasma lipid concentrations and indices of glucoregulation did not change in either group (Table 2). No differences between groups were observed for markers of inflammation, oxidative stress and glycation, and blood counts (Table 2). Serum insulin and IGF-1 concentrations did not change in either group (Table 2). Since completing the randomized trial, all but one of the participants in the SUP group continued for another 6 months on the supplementation regimen. Even with this longer, 12 month period of supplementation, no changes in any outcome were observed (data not shown).

**Table 1:** Effect of 6 months of nutritional supplements or control on indices of arterial stiffness, vasomotor function, and blood pressure.

	Supplements(n=28)	Control (n=26)	Adjusted Difference Between Groups	Between Group *P* value
Pulse wave velocity, m/s Baseline 6 months Change	5.4 ± 0.3 4.8 ± 0.3 −0.5 ± 0.4	5.2 ± 0.2 5.3 ± 0.2 0.1 ± 0.3	−0.3 ± 0.4	0.36
Augmentation index, % Baseline 6 months Change	11.7 ± 2.6 12.5 ± 2.8 0.8 ± 1.1	12.3 ± 2.8 13.0 ± 2.3 0.7 ± 1.1	0.4 ± 1.5	0.77
Flow-mediated dilation, % Baseline 6 months Change	5.0 ± 0.5 4.5 ± 0.4 −0.5 ± 0.5	4.1 ± 0.4 4.4 ± 0.3 0.3 ± 0.3	−0.3 ± 0.5	0.54
GTN-mediated dilation, % Baseline 6 months Change	16.1 ± 1.3 14.2 ± 1.6 −1.9 ± 1.1	14.6 ± 1.2 13.3 ± 0.9 −1.3 ± 1.4	−0.2 ± 1.7	0.93
Systolic BP, mmHg Baseline 6 months Change	108 ± 2 108 ± 2 0 ± 1	109 ± 2 107 ± 3 −2 ± 1	1 ± 2	0.48
Diastolic BP, mmHg Baseline 6 months Change	69 ± 1 69 ± 1 0 ± 1	66 ± 2 64 ± 2 −2 ± 1*	2 ± 1	0.08

Values are arithmetic means ± SE except for mean differences between groups, which have been adjusted for baseline values. Between-group P values reflect the between-group comparison change-scores from ANCOVAs that included baseline values as the covariate. *Significant (p≤0.05) within-group change. GTN, nitroglycerine; BP, blood pressure. Several subjects in the supplement group (n=15) and control group (n=14) did not undergo testing for GTN-mediated dilation because systolic blood pressure was below 100 mmHg, which is a contraindication to GTN administration.

**Table 2:** Risk factors for cardiovascular disease and diabetes, and circulating markers of oxidative stress, chronic inflammation, in response to 6 months of nutritional supplementation or control.

	Supplements (n=28)	Control (n=26)	Adjusted Difference Between Groups	Between Group *P* value
***Body Mass and Composition***			
Body mass, kg Baseline 6 months Change	73.8 ± 2.3 74.2 ± 2.5 0.3 ± 0.4	72.8 ± 1.8 73.1 ± 1.7 0.2 ± 0.4	0.1 ± 0.6	0.91
Body fat, % Baseline 6 months Change	30.1 ± 1.1 29.9 ± 1.0 −0.2 ± 0.3	30.2 ± 1.7 30.3 ± 1.8 0.2 ± 0.3	−0.2 ± 0.4	0.70
***Lipids***			
Triglycerides, mg/dL Baseline 6 months Change	79 ± 12 75 ± 6 −4 ± 11	78 ± 8 87 ± 8 8 ± 4	−12 ± 8	0.16
Total cholesterol, mg/dL Baseline 6 months Change	186 ± 6 186 ± 6 −1 ± 4	189 ± 6 192 ± 5 4 ± 4	−5 ± 5	0.32
LDL-cholesterol, mg/dL Baseline 6 months Change	110 ± 6 111 ± 5 1 ± 3	114 ± 6 115 ± 5 1 ± 4	−1 ± 5	0.80
HDL-cholesterol, mg/dL Baseline 6 months Change	61 ± 3 59 ± 3 −2 ± 2	61 ± 3 60 ± 3 −1 ± 2	−1 ± 2	0.59
***Glucoregulatory Function***					Fasting glucose, mg/dL Baseline 6 months Change	80 ± 2 83 ± 2 3 ± 2	84 ± 2 85 ± 2 1 ± 2	−1 ± 2	0.70
Fasting insulin, μU/mL Baseline 6 months Change	3.3 ± 0.3 3.6 ± 0.4 0.3 ± 0.3	2.8 ± 0.3 2.9 ± 0.3 0.1 ± 0.2	0.3 ± 0.4	0.41
HOMA-IR Baseline 6 months Change	0.64 ± 0.06 0.74 ± 0.09 0.10 ± 0.06	0.60 ± 0.06 0.63 ± 0.06 0.03 ± 0.04	0.08 ± 0.08	0.32
***Inflammatory Cytokines and Oxidative Stress Markers***					CRP, mg/L Baseline 6 months Change	1.69 ± 0.48 1.51 ± 0.20 −0.17 ± 0.49	1.21 ± 0.52 1.44 ± 0.56 0.23 ± 0.10*	−0.20 ± 0.43	0.46
TNFα, pg/mL Baseline 6 months Change	1.92 ± 0.09 1.94 ± 0.09 0.02 ± 0.05	2.10 ± 0.18 2.13 ± 0.19 0.03 ± 0.14	−0.05 ± 0.14	0.70
IL-6, pg/mL Baseline 6 months Change	1.26 ± 0.26 1.35 ± 0.23 0.08 ± 0.08	1.39 ± 0.31 1.68 ± 0.32 0.29 ± 0.20	−0.22 ± 0.20	0.27
Protein carbonyl, nmol/mg Baseline 6 months Change	0.83 ± 0.03 0.76 ± 0.03 −0.07 ± 0.04	0.79 ± 0.03 0.82 ± 0.03 0.03 ± 0.03	−0.06 ± 0.04	0.14
AGEs, ng/mL Baseline 6 months Change	307 ± 14 287 ± 12 −20 ± 13	272 ± 13 270 ± 12 −2 ± 12	1 ± 14	0.96
***White Cell Counts and Growth Factors***				
WBC, k/cumm Baseline 6 months Change	5.1 ± 0.2 5.0 ± 0.2 −0.1 ± 0.2	4.8 ± 0.2 5.1 ± 0.3 0.3 ± 0.2	−0.3 ± 0.3	0.20
Lymphocytes, k/cumm Baseline 6 months Change	1.50 ± 0.05 1.42 ± 0.06 −0.08 ± 0.04*	1.42 ± 0.09 1.44 ± 0.10 0.02 ± 0.05	−0.10 ± 0.06	0.11
IGF-1, ng/mL Baseline 6 months Change	145 ± 6 151 ± 7 6 ± 5	148 ± 7 144 ± 6 −4 ± 4	9 ± 6	0.15

Values are arithmetic means ± SE except for mean differences between groups which have been adjusted for baseline values. Between-group P values reflect the between-group comparison change-scores from ANCOVAs that included baseline values as the covariate. *Significant (p≤0.05) within-group change. Triglyceride data were also adjusted for a significant effect of age on the baseline to follow up changes. Within-group P values are from paired t-tests. LDL, low density lipoprotein; HDL, high density lipoprotein; HOMA-IR, homeostasis model assessment of insulin resistance; CRP, C-reactive protein, TNFα, tumor necrosis factor α; IL-6, interleukin-6; AGEs, advanced glycation end products; WBC, white blood cells; IGF-1, insulin-like growth factor-1. To convert units to SI units, multiply the conventional units by the following conversion factors: triglycerides × 0.0113 = mmol/L; total, LDL-, and HDL-cholesterol × 0.0259 = mmol/L; glucose × 0.0555 = mmol/L; insulin × 6.945 = pmol/L.

No serious adverse events occurred. Serum markers of liver and kidney function were unaffected by supplementation. Other adverse events were limited to mild gastrointestinal discomfort associated with taking the large number of oral supplements in 19% of the participants.

## DISCUSSION

Our findings indicate that daily use of multiple dietary supplements has no effect on arterial stiffness (i.e. pulse wave velocity and augmentation index), endothelial function (i.e. brachial artery flow-dependent vasodilatation) or blood pressure in nonobese men and women. Furthermore, supplementation with these compounds did not affect key metabolic variables implicated in the biology of aging, and in the pathogenesis of cardiovascular disease, including plasma markers of inflammation, oxidative stress and glycation, plasma lipids, growth factors, or body composition.

Reports from studies conducted mainly on cells in culture or in experimental animals suggest that these compounds promote metabolic health and may have anti-aging effects [4-24]. In particular, resveratrol, quercetin, curcumin, acetyl-L-carnitine, lipoic acid, fish oil, sesamin, pomegranate, cinnamon bark, and green tea extracts have all been reported to have powerful anti-oxidant and/or anti-inflammatory properties [4-24].

However, in our study we did not see any reduction in markers of inflammation or oxidative stress. Oxidative stress and inflammation are major players in the pathogenesis of arterial aging and endothelial dysfunction, which is a precursor of atherosclerosis [25-29]. Nevertheless, pulse wave velocity, augmentation index and endothelium-dependent vasorelaxation did not improve in the supplement group. Furthermore, supplementation with these compounds did not alter other well-accepted cardiometabolic risk factors, including blood pressure, insulin resistance, and serum cholesterol, triglyceride and advanced glycation end-products concentrations. Endothelial function can be improved with just a single infusion of the anti-oxidant, vitamin C [30]. Because all the supplements that we used have been reported to have powerful anti-inflammatory and anti-oxidant effects, it is interesting that no biological change in endothelial function was observed. In contrast, it has been shown that 6-mo or 1-yr supplementation with DHEA-s results in significant improvements of glucose tolerance, arterial stiffness and inflammation [31].

One possible explanation for the lack of beneficial metabolic effects of these over-the counter dietary supplements could be the low phytochemical bioavailability or inadequate supplement potency of the phytochemicals contained in some of these compounds which are available without prescription [32]. Nonetheless, the fact remains that millions of individuals in USA and Europe are consuming these supplements, sometimes instead of a healthy diet and conventional medical treatment, which might contain hazard trace amounts of pesticides and heavy metals, including lead, arsenic, mercury, and cadmium [1,33].

A placebo was not provided to control group participants in the present study, which might be viewed as a limitation. However, while a “placebo effect” might have been an explanation for significant changes in the supplement group, we did not observe changes. Therefore, in this context, the lack of a placebo is not a limitation. Moreover, although the sample size was relatively small, there was no suggestion of clinically significant differences from the between-group estimates, despite multiple comparisons. Finally, it is conceivable that some supplements might have had beneficial effects that where counteracted by negative effects of others. However, this seems unlikely and people rarely take isolated supplements, and many of these compounds are contained in foods and beverages that are consumed regularly by millions of people. Moreover, heavy supplement users believe that these compounds work better in combination, because as with food and beverages, they provide a mix of phyto-chemicals that interact and potentiate their effect (e.g. resveratrol in wine, cathechins in green and black tea, pomegranate in fruits, curcumin in spices, etc.).

Findings from the present study suggest that a combination of several popular nutritional supplements, which are commonly taken with the intention of promoting healthy longevity and preventing chronic disease, have no effects on arterial stiffness, endothelial function, inflammation, oxidative stress, and other chronic disease risk factors in non-obese men and women. Additional randomized controlled studies are still needed to assess the potential benefits of these and other dietary supplements in obese metabolically abnormal individuals.

## METHODS

### Participants

Non-obese (BMI 21.0-29.9 kg/m^2^) men and women, aged 38-65y were recruited from the Saint Louis area. Participants were free of chronic disease based on a medical history, physical examination, blood and urine chemistries, and electrocardiogram. Exclusion criteria included chronic use of medications or dietary supplements, tobacco use, alcohol abuse, and habitual vigorous exercise. Participants consented to participate in the study, which was approved by the Washington University Institutional Review Board.

### Study Design

The study was a 6-month single-blind controlled trial in which participants were randomized (1:1 ratio with stratification for sex) to a nutritional supplement (SUP) group or control (CON) group. At the end of the RCT, the participants who had been randomized to SUP continued taking the supplements for another 6-mo, while those who had been in the CON group crossed over to the supplementation regimen for 6-mo. Technicians who performed outcomes assessments were blinded to study group assignments. Participants fasted overnight (12-hr) and refrained from exercise for 24-hr before testing. For follow-up tests, participants in the SUP group were instructed to take their nutritional supplements upon waking in the morning prior to testing. The primary outcome, carotid-femoral pulse wave velocity (PWV), was measured by using Doppler flow measures. Another index of arterial stiffness, carotid artery augmentation index (AI) was measured using applanation tonometry. Endothelium-dependent and endothelium-independent brachial artery vasodilation were evaluated by using ultrasound imaging to measure flow-mediated dilation and glyceryl trinitrate- (GTN)-mediated dilation, respectively.

### Intervention

Participants in the SUP group took the following dietary supplements: resveratrol (100 mg/day), quercetin (650 mg/day),acetyl-l-carnitine HCL (500/mg/day),alpha-lipoic acid (600 mg/day), curcumin complex (900 mg/day; standardized to 95% total curcuminoids, plus piperine 5 mg), pomegranate extract (250 mg/day; standardized to 70% ellagic acid), fish oil (1 g/day, containing 300 mg of eicosapentaenoic acid and 200 mg docosahexaenoic acid), cinnamon bark (1.7 g/day), green/white/black tea complex (800 mg/day each of green, black, and white tea extract) and sesamin (1 g/day; standardized to 500 mg sesamin lignans). Sesamin was formulated by Scivation, Inc. (Burlington, NC, USA) and all others by Swanson Health Products (Fargo, ND, USA). Both groups received a daily multivitamin/mineral supplement (Daily Multi-Vitamin & Mineral, Swanson, Fargo, ND, USA) and were advised to maintain their usual diet and physical activity. Participants met with a member of the research team each month to receive supplements and to answer questions about compliance with the supplementation regimen, changes in diet, physical activity, medical conditions, and medication use, and adverse events.

### Body weight and composition

Weight and height were measured with a calibrated balance beam scale and wall-mounted stadiometer, respectively, with the participant wearing only a hospital gown and underwear. BMI was calculated (kg/m^2^). Body composition was evaluated using dual energy X-ray absorptiometry (Delphi W, software version 11.2, Hologic Corp., Waltham, MA). Bilateral brachial artery blood pressure was measured with a calibrated monitor (Dinamap 1846 SX, Critkon, Inc, Tampa, FL) according to JNC 7 guidelines.

### Circulating biomarkers of disease risk and aging

Blood samples were collected from a forearm vein into separate tubes containing sodium heparin, EDTA, and no additives. Plasma and serum were isolated by centrifugation (3500 g, 15 min, 4º C) and stored at −80º C for later batch analyses. Samples were analyzed for plasma concentrations of glucose (glucose oxidase method, 2300 Stat Plus, YSI Inc., Yellow Springs, OH) and insulin (chemiluminescence assay, Immulite 1000, Siemens USA, Malvern, PA). The homeostasis model assessment of insulin resistance (HOMA-IR) was calculated from fasting glucose and insulin. ELISA assay kits were used to measure serum concentrations of inflammatory cytokines (tumor necrosis factor α (TNFα), interleukin-6 (IL-6), C-reactive protein (CRP), Quantikine, R&D Systems, Minneapolis, MN), oxidative stress markers (protein carbonyls (Cell Biolabs, San Diego, CA) and advanced glycation end-product (AGE) N-1-carboxymethyl lysine (MBL International, Woburn, MA)), and insulin-like growth factor-1 (IGF-1, Diagnostic Systems Laboratories, Webster, TX). Plasma lipids and blood counts were measured by a CLIA-certified clinical laboratory at the medical center.

### Indices of arterial stiffness

Carotid artery augmentation index (AI) was measured by using applanation tonometry according to published guidelines [34]. Twenty digital pulse waves were recorded with a tonometer (Millar Instruments, Inc., Model #TCB-500, Houston, TX) and analyzed with Windaq software (version 2.31, DATAQ Instruments, Inc., Akron, OH). The the maximum and minimum voltage on each wave form was identified and used to calculate pulse pressure (PP). The second derivative of the pulse wave was generated and used to identify the “shoulder” on the upstroke of the raw wave form. Augmentation pressure (AP) was calculated as the difference between the peak voltage and the voltage at the shoulder. Augmentation index was calculated as AI = 100 × AP/PP for each of the 20 waveforms; the resulting values were averaged. Because AI is dependent on heart rate, AI values were adjusted to a standardized heart rate of 75 beats/min based on the inverse relationship of 4.8 AI units per 10 beats/min HR [35]. The technician visually inspected all waveforms to ensure that the landmarks had been properly identified and to omit waveforms that were of suboptimal quality due to artifacts or irregular heartbeats. When analyses were questionable (e.g. large variation in AI values among waveforms), the waveforms were re-analyzed by another technician. If discrepancies between the analyses occurred, the technicians reviewed the analyses together and if the differences could not be remediated, the data were excluded.

Pulse wave velocity (PWV) was determined according to standard procedures1 by using transcutaneous Doppler flow measurements (Model 806-CB, Parks Medical Electronics, Inc., Aloha, OR) at the right common carotid artery and the right femoral artery. Twenty Doppler wave forms were simultaneously recorded (Windaq software, version 2.31, DATAQ Instruments, Inc., Akron, OH) at the two sites. Pulse transit time was determined as the difference in pulse arrival times (based on the “foot” of the pulse wave, as determined from the second derivative of the pulse wave) for the carotid and femoral sites. Pulse transit distances between the aorta and the carotid site and the aorta and the femoral site were measured over the skin using the second intercostal space as a landmark for the aorta, with the difference between these distances being considered the pulse propagation distance [36]. PWV was calculated for each of the 20 pulses as the quotient of the propagation distance (in meters) and transit time (in seconds) and the 20 values were averaged. Quality control procedures were identical to those described above for the AI method.

### Vascular function

Brachial artery flow mediated dilation (FMD) was used to measure endothelium-dependent vasodilation and glyceryl trinitrate- (GTN)-mediated dilation was used to measure endothelium-independent vasodilation. The participant's right arm was immobilized with the shoulder abducted at 70-90º and elbow fully extended. Ultrasound images were acquired using an ultrasound system (Agilent SONOS 5500, Andover, Massachusetts) equipped with an 11-3L linear array transducer. The probe was secured using a stereotactic clamp (Noga Engineering, Ltd.) to maintain constant positioning over the artery throughout the procedure. Ultrasound images were fed to a computer for real time quantification of arterial diastolic diameter (30 ± 2 measures/sec) using Vascular Imaging Analysis software (VIA, version 9.60) [37]. Baseline diameter was recorded for 2 minutes before a pneumatic cuff (Hokanson E20 Rapid Cuff Inflator and AG101 Cuff Inflator Air Source, PMS Instruments, Ltd., Maidenhead, UK) was inflated to 200 mmHg on the right forearm to occlude blood flow. After 5 minutes of occlusion, the pressure in the cuff was rapidly released and arterial diameter was recorded continuously for 5 minutes. FMD was calculated as the percent increase in diameter from baseline to peak diameter, where baseline diameter was the average diastolic diameter over the 2-minute baseline and peak diameter was recorded as the 10-second average of the highest diastolic diameter after cuff deflation. After the FMD assessment, and after the diameter of the artery returned to baseline, a second 2-minute baseline data collection was performed, followed by administration of 0.4 mg sublingual GTN spray. Brachial artery diameter was evaluated again 5 minutes after GTN administration. GTN-mediated dilation was calculated as the percentage increase in arterial diameter from baseline to 5 minutes after GTN administration.

### Statistical Power and Analyses

Sample size estimates were calculated for PWV, as a measure of arterial stiffness and for interleukin-6 (IL-6) and C-reactive protein (CRP) as inflammatory markers. Calculations were based on data collected in our laboratory during previous studies. These studies included a randomized controlled trial on the effects of 50 mg/d oral DHEA supplementation on markers of aging in older adults [31]and cross-sectional [38] and longitudinal studies [39] on the effects of calorie restriction on markers of aging in middle-aged adults. For all sample size calculations, the alpha error rate was set at 0.05, tests were designated as two-tailed, desired power was set to 0.80, and the ratio of participants to be randomized to the intervention and no-treatment control groups was 1:1. Results indicate that 21 subjects per group would be sufficient for detecting a 3.1 m/sec improvement in pulse wave velocity, 27 subjects per groups would be sufficient for detecting a 0.86 ng/mL reduction in IL-6, and 12 subjects per groups would be sufficient for detecting a 1.60 mg/L reduction in CRP. Therefore, our proposed sample size of 56 participants (28 per group) was sufficient to detect biologically relevant changes in arterial stiffness and inflammation.

Power analyses were performed for PWV, IL-6, and CRP to determine sample size. Respective results indicated that sample sizes of 21, 27, and 12 subjects per group would be adequate for detecting significant effects. Therefore, 28 subjects per group was chosen.

Baseline characteristics were compared with independent t-tests and chi-square tests. Outcomes were analyzed with analysis of covariance, in which baseline values were included as a covariate. Paired t-tests were used for within-group paired comparisons. Significance was accepted at p≤0.05 and tests were two-tailed. Analyses were conducted with SAS for Windows XP Pro (version 9.3, SAS Institute, Cary, NC).
